# Who Could Not Avoid Exposure to High Levels of Residence-Based Pollution by Daily Mobility? Evidence of Air Pollution Exposure from the Perspective of the Neighborhood Effect Averaging Problem (NEAP)

**DOI:** 10.3390/ijerph17041223

**Published:** 2020-02-14

**Authors:** Xinlin Ma, Xijing Li, Mei-Po Kwan, Yanwei Chai

**Affiliations:** 1 College of Urban and Environmental Science, Peking University, Beijing 100871, China; maxinlin@pku.edu.cn; 2 Department of Geography and Geographic Information Science, University of Illinois at Urbana-Champaign, Urbana, IL 61801, USA; xijingl2@illinois.edu; 3 Department of Geography and Resource Management, and Institute of Space and Earth Information Science, The Chinese University of Hong Kong, Hong Kong, China; mpk654@gmail.com; 4 Department of Human Geography and Spatial Planning, Utrecht University, 3584 CB Utrecht, The Netherlands

**Keywords:** neighborhood effect averaging problem (NEAP), air pollution exposure, environmental justice, Beijing

## Abstract

It has been widely acknowledged that air pollution has a considerable adverse impact on people’s health. Disadvantaged groups such as low-income people are often found to experience greater negative effects of environmental pollution. Thus, improving the accuracy of air pollution exposure assessment might be essential to policy-making. Recently, the neighborhood effect averaging problem (NEAP) has been identified as a specific form of possible bias when assessing individual exposure to air pollution and its health impacts. In this paper, we assessed the real-time air pollution exposure and residential-based exposure of 106 participants in a high-pollution community in Beijing, China. The study found that: (1) there are significant differences between the two assessments; (2) most participants experienced the NEAP and could lower their exposure by their daily mobility; (3) three vulnerable groups with low daily mobility and could not avoid the high pollution in their residential neighborhoods were identified as exceptions to this: low-income people who have low levels of daily mobility and limited travel outside their residential neighborhoods, blue-collar workers who spend long hours at low-end workplaces, and elderly people who face many household constraints. Public policies thus need to focus on the hidden environmental injustice revealed by the NEAP in order to improve the well-being of these environmentally vulnerable groups.

## 1. Introduction

Exposure to harmful environmental factors has adverse effects on human health. Among these environmental factors, air pollution is a major public health concern [1,2]. The measurement of air pollution exposure has to rely on a particular spatial and temporal frame. Traditionally, residential-based measurement is used for long-term (or life-time) exposure assessment [3]. It is typically assumed that people who live in the same neighborhood or geographic area, such as a census tract or zip code area, are exposed to a similar level of environmental influence [4,5]. The underlying presumption is that people spend most of their time in their residential community, and thus, people who live in the same neighborhood may have the same level of exposure [6].

However, debates have about exposure assessments have increased as individuals’ mobility grows and becomes more important. There are some concerns about environmental exposure measures, especially on whether they can accurately measure individual exposures and whether the conclusions concerning environmental justice are reliable [7,8,9,10]. Nowadays, people are more and more likely to organize their lives in different places in the city. Therefore, to measure or estimate exposure to air pollution accurately can be a challenge for epidemiological studies of large populations, especially given people’s tendency to move about over time through changing pollution concentrations, and ignoring the critical role of time and human mobility in people’s exposures to environmental influences might lead to biased results [11]. Neglecting human mobility and the spatiotemporal variations of environmental pollutants can lead to inaccurate results when assessing individual exposure and health outcomes [12,13,14]. Additionally, the spatial and temporal distribution of pollutants and the existence of autocorrelations call for spatiotemporal heterogeneity models to reveal the dynamic patterns [15,16]. Methodological issues like these are manifestations of the uncertain geographic context problem (UGCoP), which mainly arises from researchers’ lack of knowledge of the true geographic context individuals are exposed to [17,18].

In addition to the UGCoP, Kwan [19] has also observed another issue that may confound research results when assessing individual exposures to environmental factors: the neighborhood effect averaging problem (NEAP). This is the phenomenon that, when people’s daily mobility is taken into account, the mobility-based exposures of those with very high or low exposures in their residential neighborhoods will tend toward the mean value of the exposure level of the participants or the population of the study area. Take air pollution exposure as an example, where people who live in the same residential neighborhood are often considered to have the same level of exposure based on data collected at stationary monitoring stations. However, when a real-time individual-based approach that takes human daily mobility into account is adopted, the exposure levels of those with very high residence-based exposures tend to be overestimated, leading to an overall tendency of an overestimation of the statistical significance and effect size of the neighborhood effect [19]. Thus, the potential impact of the NEAP on research results highlights the necessity to re-examine the accuracy of environmental injustice studies and shed new light on the environmental injustice experiences of socially disadvantaged groups.

In recent years, researchers have become aware of this measurement bias in greenspace exposure [20,21], traffic exposure [22,23], environmental impacts on mental health and substance use [24,25,26], exposure and risk misclassification in epidemiological studies [27]. Furthermore, static measurements based on people’s residential neighborhoods also seem problematic. For instance, Ma et al. found that residence-based and mobility-based exposure estimates are significantly different and concluded that whether a study observes any significant influence of an environmental factor on health may depend on what contextual units are used to assess individual exposure [28]. Kim and Kwan found that the UGCoP and the NEAP exist and may affect the results of traffic congestion exposure assessments. They argued that mobility-based exposure measures would be more accurate because they also capture individuals’ exposures in areas outside their residential neighborhoods [22]. Some scholars have started to measure environmental exposure while taking into account individuals’ daily mobility and travel routes. For instance, Wang and Kwan measured individuals’ food environment exposure based on individuals’ movement in space and time [29]. Laatikainen et al. used older adults’ spatiotemporal behavior to obtain more accurate measures of their exposure to the physical environment and its health impacts [30]. These studies attempted to obtain more accurate environmental exposure measurements and acquire a better understanding of the relationships between human health and individual exposures based on people’s daily mobility patterns.

However, up to now, it is not clear whether the NEAP equally applies to all social groups, both the advantaged groups and the vulnerable groups, and whether there are exceptions from an individual perspective. In reality, the burdens of pollution exposures tend not to be borne equally among different social groups, which often leads to environmental injustice concerns. As observed in past studies, disadvantaged social groups such as low-income people, rural laborers, or racial minorities are more vulnerable to environmental pollution when compared to privileged people [31,32,33]. As a result, low-income people tend to bear the brunt of disproportionate environmental harms because they experience higher exposures to air pollutants and other environmental risks [34,35,36]. Because members of vulnerable social groups are often victims of environmental injustice, accurate assessment of their exposures to air pollution is urgently needed in order to reveal the extent of the environmental injustice they experience. Additionally, the existence of the NEAP might depend on the context of indoor environment or outdoor environment. As an individual may spend most of their time indoors, and some studies have found that indoor and ambient outdoor pollutants have strong positive relationships [37], others find that total personal fine particle exposures were dominated by exposure to non-ambient indoor particles [38]. Therefore, it is useful to separate exposure to ambient outdoor components from non-ambient indoor ones. Furthermore, the existence of the NEAP might also depend on the context of high-pollution days and low-pollution days, as the distribution of pollutants is dynamic and environmental influences vary over space and time [39].

In this research, we seek to examine the NEAP in a high-pollution community in Beijing, China, to address the knowledge gaps mentioned above and to explain the meaning of being an exception of the NEAP from the perspective of environmental justice. Thus, the research is designed to answer two questions: (1) Can all the residents in a high-pollution community lower their residential-based exposure through mobility? (2) If not, who are the exceptions? What are the socio-economic characteristics of the vulnerable groups and how to understand their dilemma in space and time? The results might enhance our understanding of the NEAP on an individual basis and would help call our special attention to the “invisible” vulnerable groups in a specific community.

## 2. Data

### 2.1. Data and Study Area

**Figure 1:**
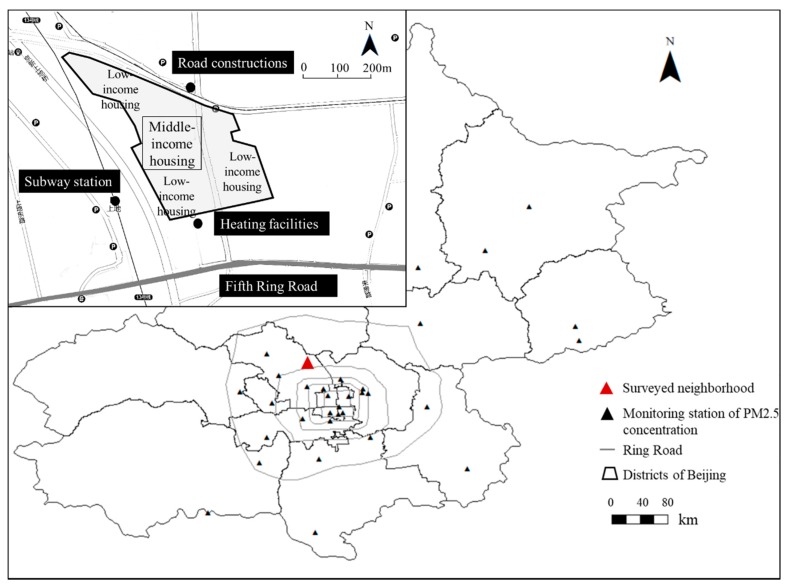
Map of the study area and sources of pollutions nearby.

We selected a high-pollution community with mixed-income residents, called Mei-he-yuan, as our study area. It is located in Beijing, China, near the Fifth Ring Road and has an area of 16 km² (Figure 1). Like other neighborhoods in suburban Beijing, this community has good access to subway and bus stations, and there are a certain amount of public facilities in the neighborhood. Residents in this residential neighborhood are able to access diverse job opportunities, including those in high-end workplaces (office buildings in the industrial park) and low-end workplaces (community property management offices usually in low-rise buildings with poor indoor environments). It is a mixed community with residents of different social-economic status. Their diverse socioeconomic backgrounds lead to diverse accessibility to high-end facilities: middle-income residents in the community with high mobility are able to access the job opportunities and use the facilities in the highly developed city center, while low-income residents with low mobility face serious constraints and have difficulty traveling to and accessing those facilities that are far away from their residential neighborhood. The different levels of daily mobility of the low- and high-income residents in the community thus lead to their diverse exposures to air pollution.

**Figure 2:**
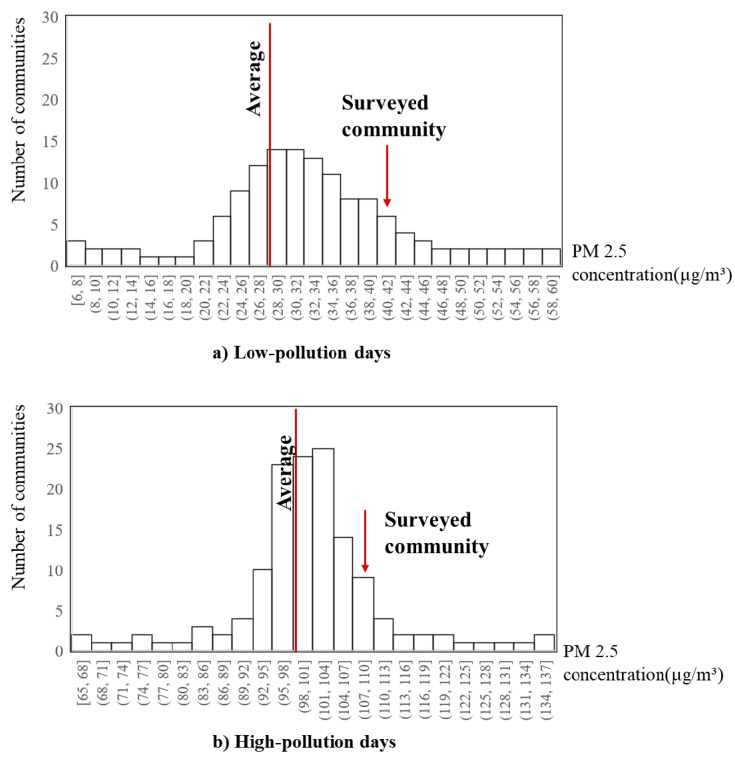
The distribution of PM_2.5_ concentrations in the communities in Beijing.(**a**) shows the average exposure of the surveyed community while the overall PM_2.5_ concentration of the whole city is below 60 μg/m^3^; (**b**) shows the average exposure of the surveyed community while the overall PM^3^ concentration of the whole city is overall 60 μg/m^3^.

Note that Mei-he-yuan is a high-pollution community in Beijing, which is under the influence of many nearby pollution sources (e.g., traffic-related pollution from the Fifth Ring Road and a subway station, chemical pollution from the heating facilities). Based on the hourly PM2.5 (particulate matter with a diameter of 2.5 μm or less) concentrations data from the monitoring stations in Beijing, this community (residential neighborhood) is one of the high-pollution communities in the city (worse than 70% of all the neighborhoods) in terms of the numbers of low-pollution and high-pollution days (see Figure 2, the PM_2.5_ concentration level is higher than 60 μg/m^3^). Its residents’ environmental exposures and the associated health threats are thus critical issues. Overall, Mei-he-yuan is a typical mixed neighborhood in suburban Beijing whose residents have diverse levels of mobility, and the community faces high environmental threats that call for an in-depth examination.

This study uses data collected in a GPS and mobile sensors-based survey in Mei-he-yuan between November 2017 and January 2018, when air pollution was very serious during the year. We used a stratified sampling approach and recruited a total of 117 residents aged 18 to 60 years old to participate in the survey via six waves. Smart air pollutant sensors, GPS tracking devices, and activity-travel diaries were used together to collect data on individuals’ space-time behaviors and real-time exposures to fine particulates (PM_2.5_). Specifically, each participant was asked to carry a GPS-equipped smartphone and a portable air pollutant sensor all the time over a continuous 48-h period that covers a workday and a weekend day (e.g., Friday and Saturday or Sunday and Monday). Moreover, each respondent was asked to complete a questionnaire and a two-day activity-travel diary that collected information about their sociodemographic attributes, activities, and travel (e.g., activity types, places visited, and travel modes used). Finally, the survey obtained valid data from 112 participants [28]. Among the 112 participants, here, we selected 106 participants from different families (we deleted six residents who had a family member participated in this research), all these 106 participants have covered all the SES (socio-economic status) types and are dependent on each other. In addition, we collected data on the hourly PM_2.5_ concentrations from the 35 stationary monitoring stations in Beijing for the corresponding survey days. These data were used to derive the residence-based exposures in this study.

Note that several tests have been done to ensure the accuracy of the individual-based real-time PM_2.5_ exposure data. First, smart air pollutant sensors are exposed to the ambient environment. It is a small portable device kept in the side pocket (fishnet bags) of a bag, thus, it can be considered as an opened bag. The bag was carried by a participant 24 h a day, which means the GPS trajectories and real-time PM_2.5_ concentrations in each participant’s immediate surroundings were recorded simultaneously at one-second intervals. The participants were monitored whether they were indoors and outdoors and whether the participants were awake and asleep. As the device is always at the hand of a participant, the data collected can be the exact figure of one’s exposure in space and time. Second, PM_2.5_ concentrations obtained by the sensors have strong correlations with each other. We did six waves of surveys and for each wave, the same devices are used to collect data. They are all of the same brand and the same model of Airbeam. The sensor logging error of Airbeam is lower than 6%; according to the United States Environmental Protection Agency in 2019 [1], it was relatively reliable among frequently-used air pollution exposure sensors. More importantly, post-hoc analysis was done to make validations after each wave of the survey. By post-hoc analysis, we found consistency between the records for each device and a high correlation between all the devices we used. After we got the data, the AirBeam PM_2.5_ readings from each individual were adjusted using calibration equations to account for inter-instrument variability (according to Krepinski K. [40]). Third, concentrations obtained from the sensors and measurements obtained from the quality-controlled monitors are strongly correlated. We did two types of experiments to make validations. One was a correlation test between the interpolation results of data from the quality-controlled monitors and the sensor data. It proved that these two datasets are correlated with each other by every minute. The other was a correlation test between sensor data on different concentration scenarios. We collected a continuous six-hour concentration dataset by the same Airbeam sensor on a low-pollution day and on a high-pollution day. It proved that these two datasets are correlated with each other. Therefore, the measurements by the Airbeam sensors in this case can be an accurate detection of one’s exposure to air pollution in space and time.

### 2.2. Sample Characteristics

In this paper, we focused on detecting the relationship between individual socioeconomic status (SES) and the “capability” of escaping from high-pollution neighborhoods through daily mobility. The socioeconomic attributes of the participants are shown in Table 1. As indicated in the table, the participants are mostly middle-aged, which means that they are in adulthood with good physical accessibility to diverse destinations. Most of them are married with at least one child, making them highly possible to shoulder the family responsibility, especially for children. 44.34% of them have private cars and thus are mobile, while others mostly use public transportation or bikes and thus have low mobility.

As the unit of analysis is the individual, we calculated income level based on reported household income divided by the number of household members, therefore, retired workers and unemployed people still got income shared by family members. Only 13.21% of the participants in the community are above Beijing residents’ average monthly income [2], but income variations still exist in this community. Note that the middle-income workers (level 3 and level 4) in the study area are more likely to work near their home, while the low-income residents tend to commute longer or work longer. With respect to occupation, the low-income workers mainly have low-end jobs such as security guards, and are thus blue-collar workers (a working-class person who performs manual labor, taking up 28.3% of the samples). On the other hand, the middle-income workers are employed in downtown Beijing or suburban employment centers, where plenty of high-end jobs like lawyers or urban planners are available. The proportion of these non-blue-collar workers (white-collar workers and unemployed ones) was 71.7% of the participants.

We also collected background information such as “Do you got an air purifier at home?”, ”Do you often smoke?” The rate of these two questions is low: 16% of the participants often smoke, and 17.9% of the participants have an air purifier at home. We took the individual’s average indoor exposure at home during a certain day into account. Therefore, if one is a heavy smoker, one’s real-time exposure at home will be very high. however, only if the person has higher exposures at some restaurants or workplaces, they will be named ”a vulnerable individual who cannot avoid exposures to a high-pollution community.” In order to detect whether a person may reduce exposure through mobility, all the home-based indoor exposures were averaged to a mean value. Pollutants at home are not explanatory factors for this paper. We only consider the influence of out-of-home pollutants.

## 3. Method

**Figure 3:**
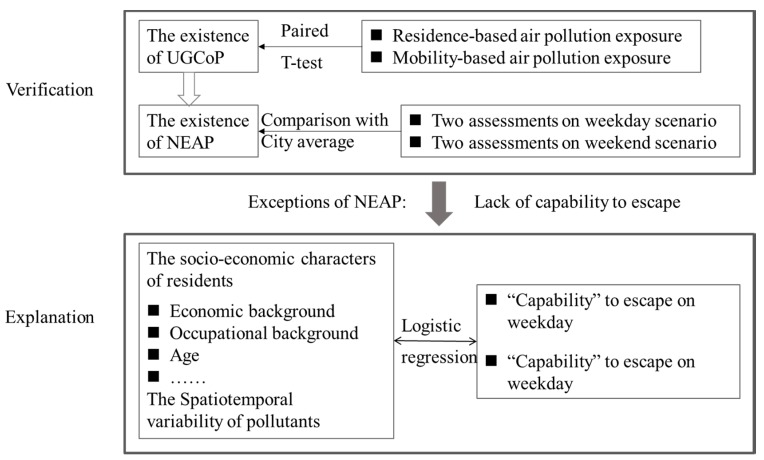
Research steps and methods. (UGCoP stands for uncertain geographic context problem, and NEAP stands for neighborhood effect averaging problem).

In order to investigate the NEAP through two measurements of air pollution exposure, we undertook the following tasks in this study (Figure 3). First, we examined whether there were manifestations of the UGCoP and the NEAP in the participants’ exposures. Second, we investigated the exceptions of the NEAP and how the exceptions (vulnerable groups) were related to participants’ daily movement. For the first part of the analysis, we used a paired sample *t*-test to examine whether mobility-based and residence-based exposures were significantly different. Then, we examined whether the NEAP existed with the help of average exposure of the city. Due to the differences in ambient PM_2.5_ concentration levels between the survey days, we divided the residence-based exposures into two groups, assigning them to either the high-pollution period (the average intraday PM_2.5_ concentration is above 60 μg/m^3^) and the low-pollution period (the average intraday PM_2.5_ concentration is below 60 μg/m^3^). We examined the two measurements under the two circumstances.

For the second part of the analysis, we applied logistic regression to explore the relationships between “whether one could avoid the high levels of pollution at the residential location” and participants’ SES. Socioeconomic characters such as income, occupation, and age were taken into consideration in order to examine which group could escape and which could not. In addition, the spatiotemporal variability of pollutants is taken into account to evaluate the results under different scenarios (i.e., high-pollution versus low-pollution period). Based on these analyses, we could get a better understanding of the experiences of the environmentally vulnerable groups in the community and their dilemma in space and time.

This study used data collected with GPS trackers (GPS trajectories are used to modify their activity diaries) and mobile pollution sensors that record individual-level, high-resolution space-time data of people’s daily movements and air pollution exposures. We used a paired sample *t*-test, which is commonly used to test the two related variables’ differences [41] and logistic regression to assess the underlying environmental injustice problem by quantifying the mobility-based and residence-based exposures of different social groups due to its advantages in interpretation [42]. After running the logistic regression model, we also used the Hosmer & Lemeshow test to validate the results by comparing with the observed value with a Chi-square test [43]. 

## 4. Results

### 4.1. Measuring Mobility-Based and Residence-Based Exposures

We use two assessment methods to measure mobility-based and residence-based individual exposures. First, the residence-based exposure levels are calculated using kriging interpolation and data from the 35 monitoring stations in Beijing. Then, we improved this residential-based measurement by dividing participants’ activities into indoor activities and outdoor ones (provided by the participants in the survey). Thus, in this study, we discussed “whether one can lower his/her residence-based mobility through mobility” and only focused on the activities carried out outside of the home.

However, we believe it is necessary to differentiate indoor exposure from outdoors. Indoor particles are very important in epidemiologic and exposure studies as people spend most of their time indoors [44]. Outdoor particles can enter indoor environments by convective flow (e.g., through an open window) or by diffusional flow (i.e., infiltration). Moreover, while indoor sources exist, indoor PM can be substantially higher than outdoor PM concentrations. In Beijing’s case, the average indoor PM_2.5_ concentration was significantly lower than that of outdoors, and the indoor PM_2.5_ was found to be mainly from the outdoors [45]. Therefore, staying indoors all day may enable a person to “escape” from residential-based exposure, and such kind of people may be wrongly excluded from the vulnerable group. Additionally, being exposed to indoor smoking or cooking may achieve momentarily higher exposure than outdoors, these people are vulnerable groups who cannot avoid exposures to home-based indoor pollution, but may be wrongly considered as members of invulnerable group with mobility.

In order to take all the circumstances into consideration, first, we got the interpolation results of the monitoring stations every second (which is C1). Second, we got the indoor concentration levels of each individual while he/she is at home (which is C2). Thus, when the participant was traveling outdoors, we used C1 as residential-based background concentration; when the participant stayed indoors, we used C2 as residential-based background concentration.

**Figure 4:**
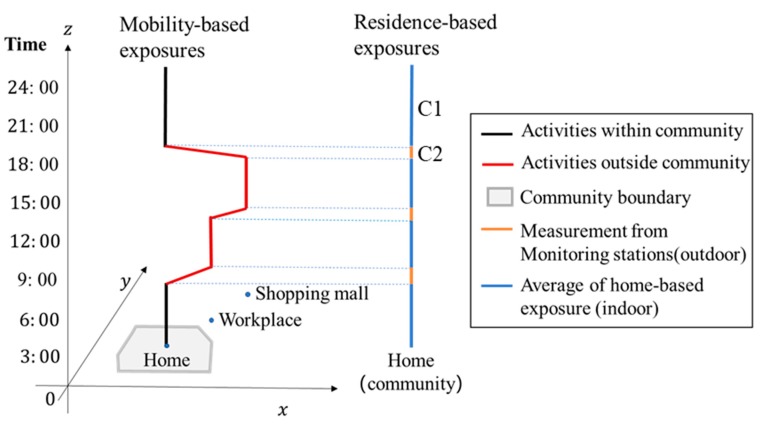
Comparison of mobility-based exposure and residence-based exposure for one participant.

As shown in Figure 4, a participant traveled to the workplace and shopping mall during the day and then returned home. They spent 13 h at home during the survey day. Thus, we got an average home-based exposure (C2) from the 13 h of indoor activities at home. The residence-based exposure for this participant is a combination of concentrations from the monitoring stations (orange) and average concentrations from their at-home indoor activities (blue). Thus, when the participant is traveling, their real-time exposure from the portable sensor will be compared with the concentration from the monitoring stations (C1). When the participant stays indoors at a workplace or shopping mall, their real-time exposure from the portable sensors will be compared with the average figure of 13-h home-based indoor exposure. In another participant’s case, if they stayed at home for 10 h on the survey day, the home-based indoor exposure will be changed accordingly.

As our data were collected on different days, both high-pollution days and low-pollution days, and participants tended to have self-adaptation in their travel behaviors if the ambient pollution level is high, their exposure might not be so high although it is in a high-pollution scenario. Therefore, in order to discuss the correlations in both cases, we have divided it into the low-pollution scenario and high pollution scenario, according to a K-means clustering algorithm (both mobility-based exposure and residential-based exposure values are considered). Then, we conducted a paired sample *t*-test to compare the measurements in the two scenarios in order to examine whether the NEAP exists.

Table 2 shows that the two measurements are significantly different from each other (*p* value less than 0.01), both on low-pollution days and high pollution days. Therefore, the measurements could be quite diverse under different contexts and the mobility context could show the interpersonal differences.

### 4.2. Examining and Visualizing Neighborhood Effect Averaging 

As postulated by the NEAP, it is assumed that residents in highly polluted communities may lower their exposures when traveling out of their residential neighborhoods. Thus, we took residential-based exposure as the “boundary line” for the study community. If the mobility-based exposure is lower than the residential-based exposure on a day, and the person has traveled out of the home during the day, it means they are able to avoid exposures to from high pollution by moving to other places. If not, they may have to travel to a worse indoor/outdoor environment; both indicate that the participant is in an environmentally venerable group.

We added the average exposure of the whole city (WC) according to the percentage of indoor household activities duration Beijing residents carried out [46], and the outdoor concentration of the whole city (WC), which is the estimation of a Beijing resident’s average exposure during a day, we may assume it as the “standard line” of the city. If the NEAP exists in the traditional measurements, then the figure of mobility-based exposure (MB) will be closer to the concentration of the whole city (WC), while the gap between residential-based exposure (RB) and WC is larger. It means the residents could lower their exposure by traveling outside the community. As shown in Table 3, the residential-based exposure in this high-pollution community is always above the exposure of the whole city. Furthermore, generally, the mean value of mobility-based exposure is lower than residential-based exposure, but are still above the average of the whole city in most cases.

**Figure 5:**
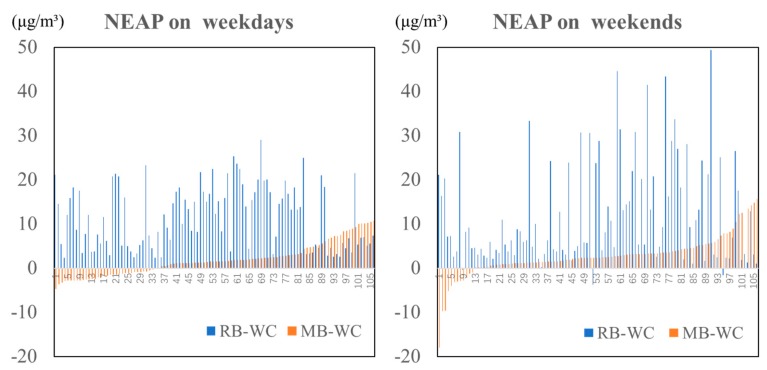
Visualization of the NEAP for the two meausremnts on weekdays and weekends.

Then we use histograms to compare the NEAP associated with the two measurements. In Figure 5, the horizontal axis is WC, while the blue bars represent the difference between RB and WC, the orange bar stands for the difference between MB and WC, and each bar indicates the deviation of one individual’s exposures (under two measurements). Although the degree of deviation from the standard exposure (WC) varies in each observation, we may find that RB-WC is always above 0, which indicates that individuals in this high-pollution community might experience higher exposure if they stay in the community for a whole day, compared to a Beijing resident’s average exposure. Additionally, it fits the description of the NEAP that, if one travels outside one’s community, one may experience exposures closer to the city standard (WC), as the heights of the orange bars are much smaller than that of the blue ones. However, there are some exceptions: some individuals could not lower their exposures through mobility. These individuals were exposed to worse environments than this high pollution community, which shows their vulnerability in terms of environmental justice. It is worthwhile to define this group in this mixed community and get to know their socio-economic characteristics.

### 4.3. Examining the Socioeconomic Factors Associated with Neighborhood Effect Averaging

As suggested earlier, there are a certain number of participants who could not lower their exposure with mobility. Thus, which social groups could not avoid exposures to the high pollution levels at their residential locations still need to be explored. Identifying the socioeconomic characteristics of these groups would help identify the vulnerable groups in this high-pollution community in the context of environmental justice. Note that residents in the mixed community of Mei-he-yuan have diverse socioeconomic characteristics. It is thus possible to distinguish the environmentally vulnerable groups among these residents and examine the factors that constrain their daily mobility relative to the privileged groups in the community.

We use logistic regression to assess whether neighborhood effect averaging is experienced differently by participants with different socioeconomic characteristics. In the model, the binary dependent variable is whether a participant’s mobility-based exposure is lower than their residence-based exposure. Its value is 1 when a participant could not lower his/her exposure through mobility (i.e., their mobility-based exposure is above their residence-based exposure), and zero otherwise (i.e., when their mobility-based exposure is equal to or lower than their residence-based exposure).

Given that neighborhood effect averaging may be related to participant’s socioeconomic characteristics, we utilized three sets of socioeconomic factors as independent variables in the logistic regression model. The first set includes respondents’ sociodemographic attributes such as age, gender, marital status and number of children. The second set represents the respondents’ economic status, including variables like income and car ownership. Car ownership not only reflects a person’s economic status but also influences their daily mobility. The last set of socioeconomic factors includes employment status (employed versus not working) and occupation (white-collar versus other jobs), which are both binary variables. White-collar jobs such as teachers, clerks, artists, designers, technicians and professors were assigned a value of 1, while other jobs such as janitors, security guards, taxi drivers and delivery persons were assigned a value of 0. Note that the average monthly income (family income divided by the number of family members) of the participants with white-collar jobs was RMB 5791, while that of the participants with blue-collar jobs was RMB 5575. Because there is no significant difference in the average monthly income between these two occupational classes, income and occupation can be included in the model because there is no correlation between them (i.e., no multicollinearity issue).

Using this logistic regression model, the relationships between socioeconomic factors and whether one could avoid residential-based high exposure (the “capability” to escape) is explored. Since work activities take up a high proportion of people’s daily activities on weekdays, and the activity patterns between weekdays and weekends are likely to be significantly different, we estimated two separate models: one is for weekdays and the other is for weekend days. The significance of the independent variables in the two models were compared, and based on that, the relationships between the selected socioeconomic characteristics and neighborhood effect averaging were examined. Additionally, whether low or high ambient pollution levels had any association with the “capability” to escape was also examined. If there was a strong association between them, the effects of the two scenarios (low-pollution period and high-pollution period) should also be discussed.

Table 4 shows the results of the logistic regression models. First, as the significance of high-pollution days is larger than 0.05, high levels of air pollution was not a significant predictor of whether mobility-based exposure could be greater than residence-based estimates or not. Second, the results show that participants’ socioeconomic characteristics have significant relationships (*p* < 0.05) with the ”capability” to escape. However, not all of the socioeconomic factors are significant and there are some differences between the two models. For weekdays, participants’ age, income, car ownership, employment status and occupation were significantly associated with the “capability”. For weekend days, participants’ age, income and occupation were significantly associated with neighborhood effect averaging.

All groups of socioeconomic factors have at least one variable that is significantly associated with the “capability” to escape for both weekdays and weekend days. Income, age and occupation were significant and had the same positive/negative relationships with the “capability” in the two models, indicating that participants with similar socioeconomic status tend to have similar activity and mobility patterns, and thus, were associated with similar patterns of neighborhood effect averaging. In this high-pollution community, the mobility-based exposures of the white-collar and middle-income participants are significantly lower than residence-based exposures, which means they might “escape” from high residence-based exposures. While the mobility-based exposures of the elderly groups tend to be higher than residence-based exposures, which means that they might fail to “escape” from high exposures near home. Compared to weekends, employment status and car ownership have significant relationships with exposure over- or underestimation on weekdays. This may be related to work-related activities which are the major daily activities for most respondents. The underlying interpretation would be elaborated in the discussion section below.

In binary logistic regression, the values of the dependent variable can be regarded as classification categories. The extent to which the classification results based on the independent variables are accurate when compared to the categories of the dependent variable in the sample can be used to evaluate the performance of the model. The result of the Hosmer & Lemeshow test indicates that the observed value of being underestimated is not significantly different from the expected value from the independent socioeconomic variables and confirms the reliability of the logistic model according to the definition of the test.

As income level is the only ordinary variable worth further discussion, we found that 15.38% of those in the income level 3 group and 28.85% of those in the income level 3 group could not avoid high exposures and experienced even higher exposures through mobility. It indicates that these groups, which share medium income among the community, may still be the environmentally vulnerable individuals with improved mobility.

These results indicate that participants’ socioeconomic characteristics such as age, income, car ownership, employment status and occupation are associated with “being exceptions of NEAP”. In the discussion section that follows, we focus on the interpretation and implications of the vulnerability of this group in everyday life activities.

## 5. Discussion

### 5.1. Neighborhood Effect Averaging as a New Dimension of Environmental Vulnerability 

Neighborhood effect averaging may be experienced by individuals in high pollution communities that lowers their exposures with the help of mobility. However, this is not always the case, and the results of this study shed light on the exceptions. Those who could not avoid exposures to residential-based high pollution are members of the low SES groups that show environmental vulnerability.

In past studies, low-income people with low mobility are often identified as the environmentally vulnerable group [47]. The exceptions of neighborhood effect averaging add a new dimension to this understanding: the ones who are struggling to make a living and taking care of families are more likely to be ”trapped” in high pollution communities. These groups may experience environmental injustice in ways that are difficult to detect. Their constraints can only be identified by examining their individual-based daily activities and travels, which may reveal that they may not be the environmentally vulnerable groups identified in traditional studies.

### 5.2. Income, Car Ownership, and Neighborhood Effect Averaging 

Income level has the highest correlation with “whether one experiences the NEAP or not”, Table 5 shows the distribution in each income group. Among the lowest-income group, the proportion of not experiencing the NEAP is very high both on weekdays and weekends. Opposite to that, those among the highest-income group could all avoid the residential-based high exposure. For the income level 2 group and income level 3 group (note that they are below the average of Beijing residents’ income), there are slight differences on weekdays and weekends. On weekends, individuals have less mandatory or obligatory activities and fewer time constraints to travel further and thus have more chances to lower their exposure through mobility.

Car ownership has a high correlation with the “capability” to escape only on weekdays. That is because on weekdays, high-income residents and low-income residents may be exposed to different ambient environments while commuting. However, on weekdays, those without cars could also travel by public transport to a better environment, while those with car tends to travel to inner-city areas with higher exposure for shopping and recreation.

### 5.3. Occupation, Age and Neighborhood Effect Averaging

Among the employed participants, blue-collar workers have similar personal income levels as white-collar workers. However, their working environments are quite different. In the surveyed community, most of the white-collar residents are lawyers, teachers or civil servants who work in high-end offices and have access to less polluted areas and workplaces than their residential neighborhood. Contrastingly, the blue-collar workers among the participants are usually delivery persons, security guards and janitors and their workplaces have poor environments such as small restaurants, security rooms and basements. As a result, they tend to spend long hours in workplaces that have high pollution, both on weekdays and weekends (some blue-collar workers work six days a week).

**Figure 6:**
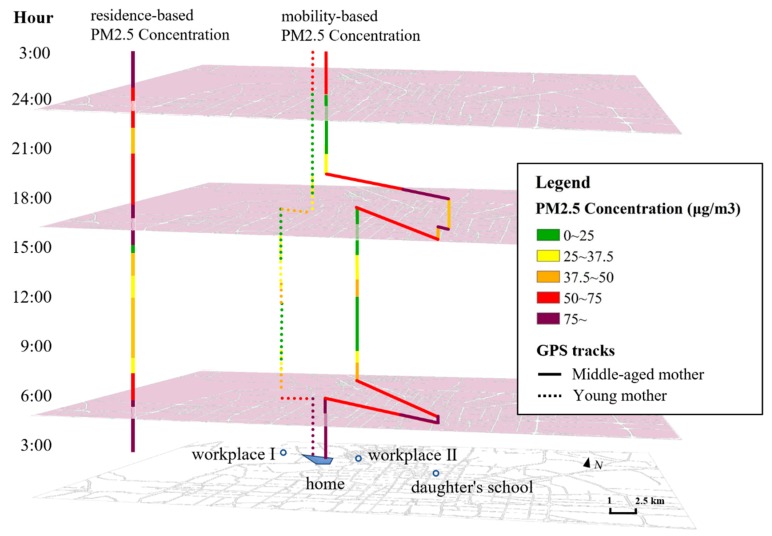
Comparison of a middle-aged mother and a young mother’s air pollution exposure.

The elderly participants are more likely to undertake more outdoor activities to take care of family members, such as grocery shopping, picking up children or visiting other elderly persons. Some of them will thus expose themselves to highly polluted air while performing these mandatory duties. As Figure 6 shows, the left column shows the PM_2.5_ concentration level of this community. It changes throughout the 24 h of a day and the highest pollution periods are from 3:00 a.m to 6:00 a.m., from 15:30 p.m.to 17:40 p.m., and from 23:00 p.m. to 3:00 a.m. The right two columns are the space-time paths of a young mother with a two-year child (left) and a middle-aged mother with a 12-year-old daughter. The middle-aged mother has to pick up her daughter and as a result is exposed to areas with higher levels of air pollution, while the young mother takes care of her child at home. Thus, the residence-based exposures of the middle-aged mother would tend to underestimate her mobility-based exposures. Therefore, residents of a high-pollution community are more likely to travel to less polluted areas in the city, which may lower the average exposure level for the day. However, middle-aged residents like her might not have the opportunity to avoid exposures to high-pollution communities both on weekdays and weekends.

We have to admit that, as the survey is carried out in one community, the estimated residence-based exposures are not generated using state-of-the-art exposure estimation methods. In order to obtain more specific estimation of localized exposure based on monitoring station data, Xu et al. [48] has adopted a large array of geographic covariates including road network information, distance to specific features, counts of restaurants and bus stops within buffers, land use mix, the normalized difference vegetation index, population density, elevation and others. These are essential for estimating the spatial distribution of pollutants. However, in our case, the residential-based exposure of residents from the same location has little difference on the same day. Therefore, land use and land cover will not be necessarily important to modify residential-based exposures.

## 6. Conclusions and Suggestions

This study found that the NEAP exists when assessing individual exposure to air pollution in a high-pollution community with two measurements. First, there are significant differences between participants’ mobility-based and residence-based exposures to air pollution. Second, neighborhood effect averaging is clearly observed for both low- and high-pollution days. With the help of mobility, the mean value of mobility-based exposure may shift toward the mean value of exposures from the entire city. Third and most importantly, whether the NEAP is associated with different individuals is affected by people’s socioeconomic characteristics such as income and employment status; however, the statistically significant socioeconomic characteristics are slightly different between weekdays and weekend days. The elderly, low-income and blue-collar participants without cars have lower mobility and are more likely to be “trapped” in high pollution neighborhoods. These environmentally vulnerable individuals are likely to be neglected using traditional residence-based exposure assessments, but stressing the NEAP may help uncover their situations. Lastly, among the factors associated with the NEAP, the spatiotemporal variability of pollutants is less important than individuals’ socioeconomic factors, which are highly related to individuals’ daily mobility.

The findings of this study highlight the problems faced by environmentally vulnerable individuals. If these problems are neglected, there will likely be aggravated environmental injustice issues. Ignoring neighborhood effect averaging may lead to serious bias in the evaluation of the environmental exposure of these vulnerable groups. Note that low-income individuals tend to have low daily mobility and could barely travel further from their residential neighborhoods. Blue-collar workers are constrained by long working hours, and their workplace environments are often worse than those of their home or residential neighborhoods. They also tend to have low accessibility to high-end facilities and fewer opportunities to lower their daily exposure levels. Finally, elderly parents who face stringent space-time constraints associated with obligatory household activities also tend to be exposed to harmful environments like road construction sites, no matter what the weather conditions and spatiotemporal variability of pollutants are.

In order to mitigate the environmental injustice revealed by examining the exceptions of the NEAP, special attention should be paid to the vulnerable groups to decrease their hidden environmental risks. Therefore, first, sophisticated facility planning is required near the neighborhood to reduce the exposure of low-income groups with low mobility. Second, more public facilities with good micro-environments such as parks should be constructed near blue-collar workers’ workplaces to increase their accessibility to better environments. Third, for elderly parents, obligatory activities in high-pollution areas should be re-distributed among all family members. It seems important to take the household perspective when attempting to reduce their high exposure to air pollution.

There are several limitations in this research that we would like to improve in future research. First, it is a case study of one high-pollution community. The results might thus be challenged by studying more types of communities at different locations. Second, to make a full use of GPS trajectories of individuals, further studies with spatial heterogeneity and temporal dynamics with high-resolution data, the detection related to the spatio-temporal autocorrelation based on Moran I or Local Indicator of Spatial Association (LISA) [49] and serial autocorrelation [50] could be introduced to reveal the complex correlation in space and time. Last but not least, the assessment of residential-based exposure needs improvement if the land use and land cover are taken into consideration, and aerosol optical depth (AOD) methods could be used to retrieve the accurate location-based exposure.

## Figures and Tables

**Table 1:** Demographic information of the participants.

Variable	Description	N	Proportion
Gender	1 (=Male)	52	49.06%
	0 (=Female)	54	50.94%
Average age		46.46	
Income level(household income divided by the number of household members)	1 (<1500 RMB);	14	13.21%
	2 (1500~4500 RMB);	52	49.06%
	3 (4500~8500 RMB);	26	24.53%
	4 (>8500 RMB)	14	13.21%
Marriage Status	1 (=Married)	72	67.92%
	0 (=Unmarried)	32	30.19%
Family structure	1 (=With at least one child)	63	59.43%
	0 (=Without children)	43	40.57%
Employment status	1 (=Employed)	82	77.36%
	0 (=Unemployed)	24	22.64%
Occupation status	1 (=Blue collar)	30	28.30%
	0 (=non-blue collar)	76	71.70%
Car ownership	1 (=with at least one car)	47	44.34%
	0 (=without cars)	59	55.66%

Note: N = 106. RMB = renminbi, the official Chinese currency.

**Table 2:** Comparison of mobility-based and residence-based exposure in different pollution senarios.

		Mobility-Based Exposure (μg/m^3^)	Residence-Based Exposure (μg/m^3^)
Low-pollution days	average	23.07	29.27
	standard deviation	11.72	12.69889417
	*p*-value of paired sample *t*-test	2.36 × 10^−11^	
	average	73.23	83.82
	standard deviation	18.23185952	20.00814382
High-pollution days	*p*-value of paired sample *t*-test	1.01 × 10^−8^	

**Table 3:** Comparison of mobility-based, residence-based exposure and the exposure of the whole city.

	Average Exposure of the Selected Community (μg/m^3^)		
	Mobility-Based	Residence-Based	Average Exposure of the Whole City (μg/m^3^)
Wave 1_weekday	65.45	71.71	62.49
Wave 2_weekday	32.19	33.89	31.44
Wave 3_weekday	34.20	38.39	32.38
Wave 4_weekday	40.30	44.20	39.65
Wave 5_weekday	37.29	41.29	35.07
Wave 6_weekday	33.02	39.52	31.19
Wave 1_weekend	10.22	11.49	9.24
Wave 2_weekend	17.01	19.57	16.25
Wave 3_weekend	18.33	19.34	17.30
Wave 4_weekend	40.17	43.21	39.65
Wave 5_weekend	110.24	114.54	109.26
Wave 6_weekend	81.06	85.94	87.63

**Table 4:** Results of the logistic regression models.

Variables	Weekday Model Parameters	Weekend Model Parameters
High-pollution days	0.197	0.319
Age	2.535 ***	2.365 **
Gender	−0.906	0.496
Marriage	0.242	−0.919
Number of Children	0.356	−3.71
Income level	−1.722 ***	−1.952 ***
Car Ownership	−1.061 *	−1.274
Employment Status	1.755 *	1.532
Occupation	1.685 **	3.112 ***
Number of observations	106	106
Model Sig.	0.000	0.000
Hosmer & Lemeshow test (Sig.)	0.468	0.753
Chi-Square	8.703	5.047

Note: * *p* < 0.05, ** *p* < 0.01, *** *p* < 0.001.

**Table 5:** The proportion of experiencing NEAP for different income level group.

	Weekdays		Weekends	
Income Level	Proportion of Experience NEAP (%)	Proportion of Not Experience NEAP (%)	Proportion of Experience NEAP (%)	Proportion of Not Experience NEAP (%)
1 (<1500 RMB)	64.29	35.71	57.14	42.86
2 (1500~4500 RMB)	63.46	36.54	71.15	28.85
3 (4500~8500 RMB)	76.92	23.08	84.62	15.38
4 (>8500 RMB)	100.00	0.00	100.00	0.00
